# Performance of Severe Acute Respiratory Syndrome Coronavirus 2 Serological Diagnostic Tests and Antibody Kinetics in Coronavirus Disease 2019 Patients

**DOI:** 10.3389/fmicb.2022.881038

**Published:** 2022-04-14

**Authors:** Hyun-Woo Choi, Chae-Hyeon Jeon, Eun Jeong Won, Seung-Ji Kang, Seung Yeob Lee, Seung-Jung Kee

**Affiliations:** ^1^Department of Laboratory Medicine, Chonnam National University Bitgoeul Hospital, Gwangju, South Korea; ^2^Department of Laboratory Medicine, Chonnam National University Hospital and Medical School, Gwangju, South Korea; ^3^Department of Laboratory Medicine, Chonnam National University Hwasun Hospital, Hwasun, South Korea; ^4^Department of Parasitology and Tropical Medicine, Chonnam National University Medical School, Gwangju, South Korea; ^5^Department of Infectious Diseases, Chonnam National University Bitgoeul Hospital, Gwangju, South Korea; ^6^Department of Infectious Diseases, Chonnam National University Medical School, Gwangju, South Korea; ^7^Department of Laboratory Medicine, Jeonbuk National University Hospital and Medical School, Jeonju-si, South Korea; ^8^Research Institute of Clinical Medicine of Jeonbuk National University, Biomedical Research Institute of Jeonbuk National University Hospital, Jeonju-si, South Korea

**Keywords:** SARS-CoV-2, antibody, performance, kinetics, binding antibody, neutralizing antibody

## Abstract

Serological testing is recommended to support the detection of undiagnosed coronavirus disease 2019 (COVID-19) cases. However, the performance of serological assays has not been sufficiently evaluated. Hence, the performance of six severe acute respiratory syndrome coronavirus 2 (SARS-CoV-2) binding antibody assays [three chemiluminescence (CLIAs) and three lateral flow immunoassays (LFIAs)] and a surrogate virus neutralization test (sVNT) was analyzed in a total of 988 serum samples comprising 389 COVID-19-positives and 599 COVID-19-negatives. The overall diagnostic sensitivities of CLIAs and LFIAs ranged from 54.2 to 56.6% and from 56.3 to 64.3%, respectively. The overall diagnostic specificities of CLIAs and LFIAs ranged from 98.2 to 99.8% and from 97.3 to 99.0%, respectively. In the symptomatic group (*n* = 321), the positivity rate increased by over 80% in all assays > 14 days after symptom onset. In the asymptomatic group (*n* = 68), the positivity rate increased by over 80% in all assays > 21 days after initial RT-PCR detection. In LFIAs, negatively interpreted trace bands accounted for the changes in test performance. Most false-positive results were weak or trace reactions and showed negative results in additional sVNT. For six binding antibody assays, the overall agreement percentages ranged from 91.0 to 97.8%. The median inhibition activity of sVNT was significantly higher in the symptomatic group than in the asymptomatic group (50.0% vs. 29.2%; *p* < 0.0001). The median times to seropositivity in the symptomatic group were 9.7 days for CLIA-IgG, 9.2 and 9.8 days for two CLIAs-Total (IgM + IgG), 7.7 days for LFIA-IgM, 9.2 days for LFIA-IgG, and 8.8 days for sVNT-IgG, respectively. There was a strong positive correlation between the quantitative results of the four binding antibody assays and sVNT with Spearman ρ-values ranging from 0.746 to 0.854. In particular, when using LFIAs, we recommend using more objective interpretable assays or establishing a band interpretation system for each laboratory, accompanied by observer training. We also anticipate that sVNT will play an essential role in SARS-CoV-2 antibody testing and become the practical routine neutralizing antibody assay.

## Introduction

Coronavirus disease 2019 (COVID-19), caused by severe acute respiratory syndrome coronavirus 2 (SARS-CoV-2), emerged in December 2019 and has become a pandemic with continued transmission (Fong et al., 2020; Wu and McGoogan, 2020). In Korea, the first COVID-19 case was confirmed in January 2020 (Korea Centers for Disease Control and Prevention, 2021). The Korean government has wisely established a strategy against COVID-19 consisting of swift implementation of *in vitro* diagnostic (IVD) devices in disease prevention and control sites, early and extensive testing using accurate real-time reverse transcriptase-polymerase chain reaction (RT-PCR) testing, systematic contact tracing, and quarantine measures (Ministry of Food and Drug Safety, 2021). Therefore, it is conceivable that the proportion of undetected patients with COVID-19 is minimal (Song et al., 2020). Nonetheless, it is possible that the undiagnosed cases, including asymptomatic patients and symptomatic patients who visit the hospital later in disease and who test negative by molecular assays, may impede the effective control of disease spread (Bae et al., 2021; Zhang et al., 2021). Hence, serological testing is recommended to support the detection of such undiagnosed cases (Guo et al., 2020). Serological testing is also essential for surveys to know the epidemic curve and set the surveillance strategy, integral to pandemic control measures. Furthermore, serologic testing helps determine antibody kinetics to predict the infection severities and outcomes in SARS-CoV-2 infection. Combined with RT-PCR, detection of the production of immunoglobulin (Ig) class can be a valuable tool to enhance sensitivity and accuracy for the detection of COVID-19. Few studies have evaluated the seroconversion of IgG or M using several commercial serologic assays (Guo et al., 2020; Orner et al., 2021).

Several types of assays have been developed for the detection of SARS-CoV-2 antibodies. As of September 9, 2021, the Korean Ministry of Food and Drug Safety approved 62 COVID-19 diagnostic reagents, including 28 PCR assays, 20 antigen assays, and 14 antibody assays (Innovative and Diagnostic Medical Device Policy Division, 2021). Among the 14 antibody assays, seven are lateral flow immunoassays (LFIAs), five are enzyme-linked immunosorbent assays (ELISAs), and two are chemiluminescent immunoassays (CLIAs). LFIAs, usually used at the point of care, detect antibodies using immunochromatographic chemistry. Manual or semiautomated 96-well ELISAs and fully automated CLIA/chemiluminescent microparticle immunoassays (CMIAs) are available to measure specific antibody subclasses such as IgA, M, and G (Zhang et al., 2021). Most SARS-CoV-2 serologic assays have been developed to target antibodies for one of the two structural proteins: the most surface-exposed spike (S) protein that comprises S1 and S2 functional subunits or the most abundantly expressed nucleocapsid (N) protein. In addition, the receptor-binding domain (RBD), which is located in the S1 subunit and mediates viral entry, is a target for detecting SARS-CoV-2 antibodies (Satarker and Nampoothiri, 2020; Tai et al., 2020; Walls et al., 2020). Antibodies can be classified into two categories according to their responses to the virus: binding antibodies (bAbs) and neutralizing antibodies (nAbs). The bAbs act against the virus-infected cells via complement activation or opsonization; on the other hand, the nAbs bind to the viral structures that block viral attachment and entry for viral replication (Klasse, 2014; Zhang et al., 2021). The gold standard for detecting nAbs against SARS-CoV-2 is the conventional plaque reduction neutralization test, for which any live pathogen and biosafety level (BSL) 3 facility are essential, making routine evaluation difficult. Recently, an ELISA-based surrogate virus neutralization test (sVNT) designed to mimic the virus-host interaction using purified RBD and angiotensin-converting enzyme 2 (ACE2) was developed, which can be performed within 1–2 h in an ordinary BSL2 laboratory (Klasse, 2014; Tan et al., 2020). However, the performance and usefulness of serological assays for detecting SARS-CoV-2 bAbs or nAbs have not yet been thoroughly assessed. Here, we evaluated the diagnostic performance of seven SARS-CoV-2 serologic assays—six bAb assays and one nAb assay. Furthermore, we investigated the dynamic characteristics of the immune responses in patients with COVID-19.

## Materials and Methods

### Sample Collection

A total of 988 serum samples were obtained from 786 patients, consisting of 199 COVID-19-positive patients confirmed using RT-PCR between March and November 2020, and 587 COVID-19-negative patients with no history of COVID-19 or any epidemiological relationship with COVID-19 between June 2019 and October 2020 at Chonnam National University Hospital (CNUH), Gwangju, South Korea. RT-PCR was performed using the PowerChek 2019-nCoV RT-PCR Kit (KogeneBiotech, Seoul, Korea). Serum remnants from blood samples retrieved for routine laboratory tests were aliquoted and stored at −80°C before the assays. This study was approved by the Institutional Review Board (IRB) of CNUH (IRB No. CNUH-2020-223). The IRB waived the requirement for informed consent because of the retrospective nature of this study.

### Severe Acute Respiratory Syndrome Coronavirus 2 Antibody Assays

Seven serological assays were assessed in this study: three CLIAs [SARS-CoV-2 IgG (Abbott, Chicago, IL, United States); Elecsys Anti-SARS-CoV-2 (Roche, Basel, Switzerland); ADVIA Centaur SARS-CoV-2 Total (Siemens, Munich, Germany)], three LFIAs [STANDARD F COVID-19 IgM/IgG Combo FIA (SD Biosensor Inc., Gyeonggi-do, Korea), briefly SDF; STANDARD Q COVID-19 IgM/IgG Combo (SD Biosensor Inc.), briefly SDQ; P4DETECT COVID-19 IgG/IgM (PRIME4DIA Co., Gyeonggi-do, Korea), briefly P4D], and one SARS-CoV-2 sVNT kit (GenScript Biotech Co., NJ, United States) (Table 1). All samples were analyzed using six SARS-CoV-2 bAb assays. Because of insufficient sample volumes, only 418 serum samples, consisting of 385 samples from COVID-19-positive patients and 33 samples from COVID-19-negative patients with false-positive results from at least one of the binding antibody assays, were subjected to the SARS-CoV-2 sVNT testing. All assays were performed at the Diagnostic Immunology Laboratory of CNUH according to the manufacturer’s instructions.

**TABLE 1:** Characteristics of SARS-CoV-2 antibody assays used in this study.

Assay	Manufacturer	Target Antibody	Antigen	Method	Analyzer	Cut-off	Sensitivity*^,†^ % (95%CI)	Specificity* % (95%CI)
SARS-CoV-2 IgG	Abbott	IgG	N	CMIA	ARCHITECT i2000SR	≥1.4 S/C	100 (95.9-100)	99.6 (99.1-99.9)
Elecsys Anti-SARS-CoV-2	Roche	Total (IgM + IgG)	N	ECLIA	Cobas e801	≥1.0 COI	99.5 (97.0-100)	99.8 (99.7-99.9)
ADVIA Centaur SARS-CoV-2 Total	Siemens	Total (IgM + IgG)	RBD in S1	CLIA	Centaur XP	≥1.0 S/CO	98.7 (93.2-99.8)	99.8 (99.5-99.9)
STANDARD F COVID-19 IgM/IgG Combo FIA	SD BIOSENOR	IgM, IgG (separately)	N + S	LF-FIA	STANDARD F2400	≥1.0 COI	98.9 (93.8-99.9)	90.6 (85.0-94.7)
STANDARD Q COVID-19 IgM/IgG Combo	SD BIOSENOR	IgM, IgG (separately)	N + S	LFIA	Manual	−	96.9 (91.3-99.4)	95.7 (92.3-97.9)
P4DETECT COVID-19 IgM/IgG	PRIME4DIA	IgM, IgG (separately)	N + S1	LFIA	Manual	−	96.7 (82.8-99.9)	100 (88.4-100)
SARS-CoV-2 Surrogate Virus Neutralization Test	GenScript	IgG (nAb)	RBD in S1	ELISA	ThunderBolt	≥30%I**^‡^**	100 (87.1-100)	100 (95.8-100)

*Manufacturer specified sensitivity and specificity in each assay kit insert.

*^†^Sensitivity was based on samples collected ≥ 14 days after RT-PCR positive or symptom onset. ^‡^Percentage inhibition (%I) = [1 – (sampled optical density value/negative control optical density value)] × 100. CI, confidence interval; CLIA, chemiluminescence immunoassay; CMIA, chemiluminescent microparticle immunoassay; COI, cut-off index; COVID-19, coronavirus disease 2019; ECLIA, electrochemiluminescence immunoassay; IgG, immunoglobulin G; IgM, immunoglobulin M; LFIA, lateral flow immunoassay; LF-FIA, lateral flow fluorescence immunoassay; N, nucleocapsid protein; nAb, neutralizing antibody; RBD, receptor-binding domain; S1, subunit 1 of spike protein; SARS-CoV-2, severe acute respiratory syndrome coronavirus 2; S/C, sample/calibrator.*

### Statistical Analysis

The sensitivity [true positive/(true positive + false negative)], specificity [true negative/(false positive + true negative)], positive predictive value [PPV: sensitivity × prevalence/ (sensitivity × prevalence + (1 - specificity) × (1 − prevalence))], negative predictive value [NPV: specificity × (1 − prevalence)/((1 − sensitivity) × prevalence + specificity × (1 − prevalence))], and accuracy [sensitivity × prevalence + specificity × (1 - prevalence)] for the three CLIAs and three LFIAs were calculated based on RT-PCR results and the history of COVID-19 or epidemiological relationship with COVID-19. For LFIAs, the separated and combined results of IgM and IgG were included in the performance analysis. The detection rates of SARS-CoV-2 antibody assays in known COVID-19-positive samples were assessed based on the number of days post symptom onset in the symptomatic group and the number of days post the initial positive RT-PCR detection in the asymptomatic group. The degree of agreement between assays was quantified using the agreement percentage and Cohen’s kappa (κ) value and further evaluated by McNemar’s test of asymmetry (McHugh, 2012; Perkmann et al., 2020). The Fisher’s exact test was performed to calculate *p*-values for differences in proportions between assays. Normality tests were performed using the D’Agostino-Pearson test. The Mann–Whitney *U*-test was used to compare assay level results based on the number of days post symptom onset. The Spearman correlation coefficient (ρ) was used to measure the strength and direction of the correlation between four bAb assays vs. sVNT (Laerd Statistics, 2020). Statistical significance was set at *p* < 0.05. All statistical analyses were performed using the MedCalc Diagnostic Test Evaluation Calculator^1^ and GraphPad Prism software (version 5.03).

## Results

### Study Population and Sample Characteristics

The clinical and demographic characteristics of the patients and samples used in this study are summarized in Table 2. Of a total of 988 serum samples, 389 (39.4%) were obtained from 199 COVID-19-positive patients [59.8% female; median age (IQR), 56 (38–67) years], whereas 599 (60.6%) were obtained from 587 COVID-19-negative patients [62.4% female; median age (IQR), 54 (38–68) years]. Of the 389 samples from COVID-19-positive patients, single samples were from 102 (51.3%) patients, and multiple samples were from 97 (48.7%) patients: two samples were from 48 patients; three from 27 patients; four from 12 patients; five from six patients; six from one patient; eight from one patient; and nine from two patients. The multiple samples from one patient were serially collected at different time points, showing that about one or two samples per week were collected as follows: the first samples (patient number = 97) were collected at 2 days post the diagnosis of COVID-19; the second (*n* = 97) at 9 days; the third (*n* = 49) at 12 days; the fourth (*n* = 22) at 17 days; the fifth (*n* = 10) at 18.5 days; the sixth (*n* = 4) at 16 days; the seventh (*n* = 3) at 19 days; the eighth (*n* = 3) at 23 days; and the ninth (*n* = 2) at 28.5 days (Supplementary Table 1). The median number of multiple samples given by the same patient was three. Of the 389 positive samples, 321 (82.5%) and 68 (17.5%) were obtained from 158 symptomatic and 41 asymptomatic COVID-19 patients, respectively. Of the 599 samples from COVID-19-negative patients, most (96.5%) were one sample per patient; 144 (24%) were collected during the pre-pandemic period from June 2019 to November 2019, which were all antinuclear antibody (ANA)-positive; and 455 (76%) were collected during the pandemic period from December 2019 to October 2020, consisting of 88 ANA-positive, 340 viral-infected or positive for antibodies other than SARS-CoV-2, and 27 bacterial or parasite antibody-positive.

**TABLE 2:** Characteristics of samples tested for SARS-CoV-2 antibody assays in this study.

Characteristics	Patients	Samples
Total number, n	786	988
Female/male, n	485/301	
Age, median (IQR), year	55 (38–68)	
**COVID-19 positive patients, n (%)**	199 (25.3)	389 (39.4)
Female/male, n	119/80	
Age, median (IQR), year	56 (38–67)	
Patient with given multiple samples, n (%)		
1 sample	102 (51.3)	102 (26.2)
2 samples	48 (24.1)	96 (24.7)
3 samples	27 (13.6)	81 (20.8)
4 samples	12 (6.0)	48 (12.3)
5 samples	6 (3.0)	30 (7.7)
6 samples	1 (0.5)	6 (1.5)
8 samples	1 (0.5)	8 (2.1)
9 samples	2 (1.0)	18 (4.6)
Symptomatic (Days after the onset of symptoms), n (%)	158 (79.4)	321 (82.5)
1–7 days		98 (30.5)
8–14 days		111 (34.6)
15–21 days		59 (18.4)
22–28 days		27 (8.4)
≥ 29 days		26 (8.1)
Asymptomatic (Days after initial RT-PCR detection), n (%)	41 (20.6)	68 (17.5)
1–7 days		47 (69.1)
8–14 days		13 (19.1)
15–21 days		5 (7.4)
22–28 days		1 (1.5)
≥ 29 days		2 (2.9)
**COVID-19 negative patients, n (%)**	587 (74.6)	599 (60.6)
Female/male, n	366/221	
Age, median (IQR), year	54 (38–68)	
Patient with given multiple samples, n (%)		
1 sample	578 (98.5)	578 (96.5)
2 samples	7 (1.2)	14 (2.3)
3 samples	1 (0.2)	3 (0.5)
4 samples	1 (0.2)	4 (0.7)
Pre-pandemic (Before December 2019)		144 (24.0)
ANA-positive		144 (100)
Pandemic (Since December 2019)		455 (76.0)
ANA-positive		88 (19.3)
Viral antibody-positive		340 (74.7)
Bacterial or parasite antibody-positive		27 (5.9)

*ANA, antinuclear antibody; COVID-19, coronavirus disease 2019; IQR, interquartile range; n, number; RT-PCR, reverse transcription-polymerase chain reaction; SARS-CoV-2, severe acute respiratory syndrome coronavirus 2.*

### Overall Diagnostic Performance of Severe Acute Respiratory Syndrome Coronavirus 2 Antibody Assays

The diagnostic performance of SARS-CoV-2 bAb assays is described in Table 3. The diagnostic sensitivity of the CLIAs ranged from 54.2 to 56.6%, with no significant difference between the assays (*p* > 0.05). The sensitivity of the LFIAs ranged from 56.3 to 64.3%, showing a significant difference between SDF and P4D (*p* = 0.0279). SDF showed the highest sensitivity among the six assays, which was significantly different from all the other assays except SDQ. The diagnostic specificity of the CLIAs ranged from 98.2 to 99.8%, with a significant difference between Roche and Siemens (*p* = 0.0061). The specificity of the LFIAs ranged from 97.3% to 99.0%, without any significant difference between the assays. There was no significant difference in the pooled sensitivity and specificity between CLIAs and LFIAs. The Roche had the highest PPV (84.9%), whereas the SDQ had the lowest (28.5%). The NPV of each assay was comparable, ranging from 99.2% to 99.4%. The accuracy of the CLIAs ranged from 97.5% to 99.1%, and that of the LFIAs ranged from 96.7% to 98.3%.

**TABLE 3:** Diagnostic performance of SARS-CoV-2 antibody assays according to days after symptom onset.

	Abbott	Roche	Siemens	CLIA	SDF			SDQ			P4D			LFIA	sVNT^†^	
	IgG	Total	Total	pooled	IgM	IgG	IgM/ IgG	IgM	IgG	IgM/ IgG	IgM	IgG	IgM/ IgG	pooled	IgG	%I^‡^
Overall diagnostic performance*																
Sensitivity, %	55.3	54.2	56.6	55.4	58.9	54.8	64.3	56.0	55.0	61.7	49.9	47.8	56.3	59.3	62.3	44.3
Specificity, %	99.5	99.8	98.2	99.2	98.5	99.2	97.7	97.7	99.7	97.3	99.2	99.8	99.0	98.5	−	−
PPV, %	65.6	84.9	34.8	61.8	40.4	53.2	32.2	29.3	74.0	28.5	50.8	83.2	49.3	41.9	−	−
NPV, %	99.2	99.2	99.2	99.2	99.3	99.2	99.4	99.2	99.2	99.3	99.1	99.1	99.2	99.3	−	−
Accuracy, %	98.8	99.1	97.5	98.5	97.8	98.4	97.1	97	98.9	96.7	98.3	99	98.3	97.8	−	−
**Sensitivity in positive COVID-19 samples**																
***Symptomatic, days post symptom onset***																
Total (*n* = 321),%	60.4	59.2	59.8	59.8	62.3	60.1	68.2	59.2	59.8	65.7	53.6	54.2	61.1	56.3	65.6	50.0
1–7 days (*n* = 98),%	23.5	22.5	22.5	22.8	33.7	26.5	36.7	28.6	22.5	30.6	22.5	21.4	26.5	27.7	32.3	20.3
8–14 days (*n* = 111),%	59.5	57.7	58.6	58.6	64.9	60.4	68.5	61.3	60.4	65.8	56.8	53.2	61.3	61.4	64.6	46.8
15–21 days (*n* = 59),%	91.5	89.8	91.5	90.9	91.5	88.1	96.6	89.8	91.5	96.6	86.4	88.1	94.9	91.5	94.9	85.2
22–28 days (*n* = 27),%	100	96.3	96.3	97.5	85.2	92.6	96.3	85.2	88.9	96.3	77.8	85.2	92.6	88.9	96.3	89.5
≥ 29 days (*n* = 26),%	92.3	96.2	96.2	94.9	69.2	88.5	92.3	69.2	96.2	96.2	57.7	73.1	80.8	80.4	96.0	81.0
***Asymptomatic, days after initial RT-PCR detection***																
Total (*n* = 68),%	30.9	30.9	41.2	34.3	42.7	29.4	45.6	41.2	32.4	42.7	32.4	17.7	33.8	35.3	47.1	29.2
1–7 days (*n* = 47),%	12.8	12.8	27.7	17.8	21.3	12.8	23.4	23.4	12.8	23.4	19.2	10.6	21.3	18.7	25.5	15.1
8–14 days (*n* = 13),%	69.2	69.2	69.2	69.2	92.3	61.5	92.3	84.6	76.9	84.6	61.5	30.8	61.5	71.8	92.3	45.5
15–21 days (*n* = 5),%	60.0	60.0	60.0	60.0	100.0	60.0	100.0	80.0	60.0	80.0	60.0	20.0	60.0	68.9	100	54.4
22–28 days (*n* = 1),%	100	100	100	100	0.0	100	100	100	100	100	0.0	0.0	0.0	55.6	100	30.9
≥ 29 days (*n* = 2),%	100	100	100	100	100	100	100	100	100	100	100	100	100	100	100	56.1
**Specificity in negative COVID-19 samples**																
Pre-pandemic (*n* = 144), % (n)	100	100	99.3	−	97.9	98.6	96.5	97.2	100	97.2	98.6	100	98.6	−	−	−
False positive, n	0	0	1	−	3	2	5	4	0	4	2	0	2	−	−	−
Pandemic (*n* = 455), % (n)	99.3	99.8	97.8	−	98.7	99.3	98.0	97.8	99.6	97.4	99.3	99.8	99.1	−	-	−
False positive, n	3	1	10	−	6	3	9	10	2	12	3	1	4	−	−	−

*Since the PPV, NPV, and accuracy are dependent on disease prevalence, the rate of the accumulated confirmed cases of COVID-19 in South Korea, 1.7% (on July 2021), was counted as disease prevalence for the calculation.

*^†^Only 385 samples from COVID-19 positive patients were evaluated with the SARS-CoV-2 sVNT. Among the 321 samples from symptomatic patients, the sVNT was available only in 317 samples because of the limited sample volume: 1–7 days (n = 96), 8–14 days (n = 110), 15–21 days (n = 59), 22–28 days (n = 27), ≥ 29 days (n = 25). ^‡^Median percentage inhibition. Abbott, SARS-CoV-2 IgG (Abbott); CLIA, chemiluminescent immunoassay; COVID-19, coronavirus disease 2019; LFIA, lateral flow immunoassay; n, number; NPV, negative predictive value; %I, percentage inhibition = [1 – (sampled optical density value/negative control optical density value)] × 100; PPV, positive predictive value; P4D, P4DETECT COVID-19 IgM/IgG (PRIME4DIA); Roche, Elecsys Anti-SARS-CoV-2 (Roche); RT-PCR, reverse transcription-polymerase chain reaction; SARS-CoV-2, severe acute respiratory syndrome coronavirus 2; SDF, STANDARD F COVID-19 IgM/IgG Combo FIA (SD BIOSENSOR); SDQ, STANDARD Q COVID-19 IgM/IgG Combo (SD BIOSENSOR); Siemens, ADVIA Centaur SARS-CoV-2 Total (Siemens).*

### Interpretation of Trace Bands in Lateral Flow Immunoassay

The relatively strong and weak bands observed in SDQ and P4D were interpreted as distinctively positive and trace bands, respectively, by visual observation, whereas those in SDF were presented as index values using the fluorescence-based automated analyzer. Three observers reached a consensus through discussion of the trace bands observed in the SDQ and P4D. We compared the SDF index values between distinct positive and trace bands in SDQ and P4D according to the antibody type (Table 4). The proportion of IgM trace bands was significantly lower in SDQ than that in P4D (19.3% vs. 27.8%, *p* = 0.0471), whereas the proportion of IgG trace bands was comparable between SDQ and P4D (6.5% vs. 11.3%, *p* = 0.1109). In both assays, the trace bands were more frequently detected for IgM compared with IgG (19.3% vs. 6.5%, *p* < 0.0001 for SDQ; 27.8% vs. 11.3%, *p* < 0.0001 for P4D, respectively). The mean SDF indices were significantly lower in samples with trace bands compared to those with distinctively positive bands in SDQ and P4D (mean ± SD, 2.29 ± 1.23 vs. 7.12 ± 3.37, *p* < 0.0001 for SDQ-IgM; 3.80 ± 5.13 vs. 15.01 ± 4.83, *p* < 0.0001 for SDQ-IgG; 3.41 ± 1.82 vs. 7.99 ± 3.11, *p* < 0.0001 for P4D-IgM; and 9.50 ± 6.44 vs. 16.31 ± 3.11, *p* < 0.0001 for P4D-IgM, respectively). If the trace bands were considered negative, the sensitivity of SDQ-IgM and P4D-IgM would have decreased significantly (56.0% vs. 45.2%, *p* = 0.0033; 49.9% vs. 36.0%, *p* = 0.0001, respectively), and the specificity of SDQ-IgM would have increased significantly (97.7% vs. 99.7%, *p* = 0.0039).

**TABLE 4:** The comparison of the distinct positive bands and the trace bands in LFIAs by visual reading in compliance to the index of LFIA by fluorescence-based automated reading.

	SDQ		P4D	
	IgM	IgG	IgM	IgG
Confirmed as positive, N	218	214	194	186
**Distinct positive band**				
N	176	200	140	165
%	80.7	93.5	72.2	88.7
SDF index (COI), mean ± SD	7.12 ± 3.37	15.01 ± 4.83	7.99 ± 3.11	16.31 ± 3.11
**Trace band**				
N	42	14	54	21
%	19.3	6.5	27.8	11.3
SDF index (COI), mean ± SD	2.29 ± 1.23	3.80 ± 5.13	3.41 ± 1.82	9.50 ± 6.44
*p-*value	< 0.0001	< 0.0001	< 0.0001	< 0.0001
**By reclassifying “trace” as “negative”**				
Sensitivity, %	45.2	51.4	36	42.4
Specificity, %	99.7	100	100	99.8

*COI, cut-off index; LFIA, lateral flow immunoassay; N, n, number; P4D, P4DETECT COVID-19 IgM/IgG (PRIME4DIA); SD, standard deviation; SDF, STANDARD F COVID-19 IgM/IgG Combo FIA (SD BIOSENSOR); SDQ, STANDARD Q COVID-19 IgM/IgG Combo (SD BIOSENSOR).*

### Sensitivity of Severe Acute Respiratory Syndrome Coronavirus 2 Antibody Assays at Different Time Stages With and Without Symptoms

**FIGURE 1:**
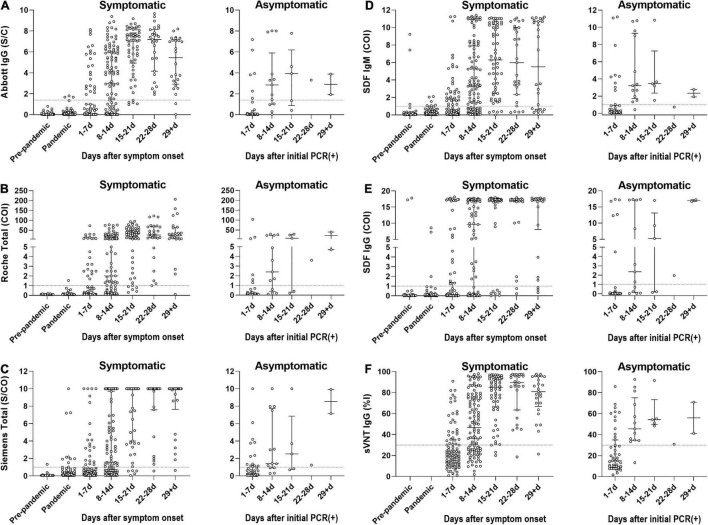
The diagnostic sensitivities of SARS-CoV-2 antibody immunoassays in 389 samples from COVID-19-positive patients according to the presence or absence of symptoms and different time stages. The 321 samples from symptomatic COVID-19 patients were subdivided according to the number of days post symptom onset. The 68 samples from asymptomatic COVID-19 patients were subdivided based on days after initial RT-PCR detection. **(A)** Seropositivity of Abbott SARS-CoV-2 IgG (Abbott IgG). **(B)** Seropositivity of Roche Anti-SARS-CoV-2 (Roche Total). **(C)** Seropositivity of Siemens SARS-CoV-2 Total (Siemens Total). **(D)** Seropositivity of IgM of STANDARD F COVID-19 IgM/IgG Combo FIA (SDF IgM). **(E)** Seropositivity of IgG of STANDARD F COVID-19 IgM/IgG Combo FIA (SDF IgG). **(F)** Seropositivity of SARS-CoV-2 Surrogate Virus Neutralization Test (sVNT). The circle represents an individual sample. The dotted line indicates the cut-off value of each assay. The horizontal lines in scattered circles represent the median value with the interquartile range. COI, cut-off index; %I, percentage inhibition; PCR, polymerase chain reaction; S/C, sample/calibrator; S/CO, sample/cut-off.

The diagnostic sensitivities of the six bAb assays in 389 samples from COVID-19-positive patients according to the presence or absence of symptoms and different time stages are shown in Table 3 and Figures 1A–E. The 321 samples from symptomatic COVID-19 patients were subdivided according to the number of days post symptom onset as follows: 1–7 days, 98 (30.5%) sera; 8–14 days, 111 (34.6%) sera; 15–21 days, 59 (18.4%) sera; 22–28 days, 27 (8.4%) sera; and ≥ 29 days, 26 (8.1%) sera. The 68 samples from asymptomatic COVID-19 patients were subdivided based on days after initial RT-PCR detection as follows: 1–7 days, 47 (69.1%) sera; 8–14 days, 13 (19.1%) sera; 15–21 days, 5 (7.4%) sera; 22–28 days, 1 (1.5%) sera; and ≥ 29 days, 2 (2.9%) sera. In the symptomatic group, the sensitivities of all six serological assays increased over 80% > 14 days after symptom onset. In the asymptomatic group, the sensitivities of both SDF and SDQ reached over 80% 8–14 days after initial RT-PCR detection, while those of the other assays reached over 80% > 21 days. In LFIAs, in the first 2 weeks of illness, the sensitivity of IgM was higher than that of IgG. The sensitivity of IgG began to exceed that of IgM after 15 days and was completely reversed after over 29 days for all LFIAs.

A total of 385 samples from 196 COVID-19-positive patients were evaluated using the SARS-CoV-2 sVNT (Table 3 and Figure 1F). The sVNT quantifies the inhibitory activity of the RBD-targeting nAbs, and the result is expressed as percentage inhibition (%I) = [1 – (sampled optical density value/negative control optical density value)] × 100. The median value of the nAb inhibition activity of the total COVID-19 samples was 44.3%. The median inhibition activity was significantly higher in the symptomatic group than in the asymptomatic group (50.0% vs. 29.2%; *p* < 0.0001). At 1–7 days after symptom onset, 32.3% were positive, with a median inhibition activity of 20.3% (cut-off: 30%). At 8–14 days, 64.6% were positive, with a median inhibition activity of 46.8%. At 15–21 days, 94.9% were positive, with a median inhibition activity of 85.2%. At 22–28 days, 96.3% were positive, with a median inhibition activity of 89.5%. After ≥ 29 days, the positive rate was 96.0%, and the inhibition activity was 81.0%.

### Specificity and False-Positive Results in Coronavirus Disease 2019-Negative Samples

The specificities of the three CLIAs and three LFIAs in COVID-19-negative samples are described in Table 3. The overall false-positive rate of the CLIAs ranged from 0.2 to 1.8%, and that of the LFIAs ranged from 0.2 to 2.7%. Among a total of 599 COVID-19-negative samples, a total of 34 samples were found to be false-positive for at least one of six serologic assays (Supplementary Table 2). Of those 455 COVID-19-negative samples collected during the pandemic period, 27 showed false-positive (5.9%). Of those 144 COVID-19-negative samples collected in the pre-pandemic period, seven showed false-positive (4.9%). Fisher’s exact test showed no significant difference in proportions between the two groups (*p* = 0.8363). Among the 34 discordant (false-positive) samples, 20 (58.8%) showed false-positive results in one assay, 11 (32.4%) in two assays, and 3 (8.8%) in three assays. For CLIAs, the three false-positive results using the Abbott were weakly positive, so was the single false-positive result using the Roche. The 11 false-positive results obtained using the Siemens exhibited a wide range of positivity, from weak to strong. For LFIAs, IgM demonstrated more false-positive results than IgG. Using SDF, 42.9% (6/14) of the false-positive results were weakly positive. Using SDQ, 87.5% (14/16) were weakly positive (trace band), and using P4D, 83.3% (5/6) were weakly positive (trace band). To validate the 34 false-positive samples, we performed additional sVNT, except for one sample (No. 608) due to insufficient sample volume. Among the 33 validated samples, only one (sample No. 501) was weakly positive for sVNT (cut-off: 30% inhibition). The sample No. 501, positive only for SDF-IgG, had an ANA-positive feature and was obtained during the pre-pandemic period.

### Agreement Between the Severe Acute Respiratory Syndrome Coronavirus 2 Antibody Assays

The overall/positive/negative percent agreement, Cohen’s κ-values, and McNemar’s test of asymmetry between the six SARS-CoV-2 antibody assays are presented in Table 5 and Supplementary Table 3. The agreement percentages ranged from 91.0 to 97.8%, with the κ-values ranging from 0.734 to 0.935. For CLIAs, Abbott and Roche showed the highest agreement rate (97.4%, κ = 0.923), whereas, for LFIAs, SDF-IgG and SDQ-IgG showed the highest agreement rate (97.8%, κ = 0.935). Comparing the agreement rates of LFIAs with those of CLIAs, the IgG of all LFIAs showed the highest agreement rate with Abbott-IgG (SDF: 96.4%, κ = 0.894; SDQ: 97.0%, κ = 0.911; and P4D: 94.8%, κ = 0.842). Despite a good or very good overall inter-assay agreement, significant differences were shown using McNemar’s test between CLIA and LFIA (in particular, SDF and SDQ) (Table 5).

**TABLE 5:** Agreement rate analysis between the SARS-CoV-2 antibody assays using agreement percentage (%), Cohen’s kappa (κ), and McNemar’s test.

% (κ) *p*-value*	Abbott- IgG	Roche- Total	Siemens-Total	SDF- IgM	SDF- IgG	SDF- IgM/ IgG	SDQ- IgM	SDQ- IgG	SDQ- IgM/ IgG	P4D- IgM	P4D- IgG	P4D- IgM/ IgG
Abbott-IgG												
Roche-total	97.4 (0.923) 0.3268											
Siemens-total	94.0 (0.830) 0.1182	94.2 (0.834) 0.0171										
SDF-IgM	93.3 (0.812) 0.0193	92.5 (0.787) 0.0037	92.0 (0.779) 0.4996									
SDF-IgG	96.4 (0.894) 0.8676	96.0 (0.787) 0.4292	94.0 (0.830) 0.1182	92.7 (0.795) 0.0251								
SDF-IgM/IgG	93.9 (0.836) < 0.0001	93.3 (0.818) < 0.0001	92.8 (0.809) < 0.0001	97.4 (0.931) < 0.0001	95.3 (0.874) < 0.0001							
SDQ-IgM	93.7 (0.822) 0.0987	93.1 (0.803) 0.0212	92.4 (0.788) 1.0000	96.8 (0.911) 0.3768	93.3 (0.810) 0.1096	96.8 (0.911) < 0.0001						
SDQ-IgG	97.0 (0.911) 0.8551	96.4 (0.893) 0.6171	94.2 (0.835) 0.0637	92.1 (0.777) 0.0174	97.8 (0.935) 0.8312	93.9 (0.835) < 0.0001	92.1 (0.777) 0.0608					
SDQ-IgM/IgG	94.7 (0.856) < 0.0001	94.1 (0.838) < 0.0001	93.2 (0.818) 0.0034	94.5 (0.854) 0.0207	94.7 (0.856) < 0.0001	96.4 (0.906) 0.2433	97.6 (0.935) < 0.0001	94.5 (0.854) < 0.0001				
P4D-IgM	92.8 (0.784) 0.0327	92.6 (0.776) 0.1602	91.9 (0.763) 0.0005	94.8 (0.850) < 0.0001	92.8 (0.784) 0.0327	92.8 (0.801) < 0.0001	96.7 (0.902) < 0.0001	92.6 (0.777) 0.0611	94.8 (0.850) < 0.0001			
P4D-IgG	94.8 (0.842) < 0.0001	94.6 (0.834) 0.0010	92.9 (0.788) < 0.0001	91.0 (0.734) < 0.0001	96.3 (0.885) < 0.0001	91.6 (0.764) < 0.0001	92.4 (0.774) < 0.0001	96.3 (0.885) < 0.0001	92.6 (0.789) < 0.0001	91.0 (0.734) 0.1691		
P4D-IgM/IgG	94.4 (0.840) 0.4185	94.2 (0.833) 0.1120	93.1 (0.806) 0.5443	94.4 (0.845) 0.1056	95.0 (0.857) 0.3914	95.0 (0.867) < 0.0001	96.3 (0.895) 0.3239	94.8 (0.851) 0.2626	96.5 (0.904) < 0.0001	97.4 (0.922) < 0.0001	94.4 (0.845) < 0.0001	

*Calculated using McNemar’s test. Abbott, SARS-CoV-2 IgG (Abbott); P4D, P4DETECT COVID-19 IgM/IgG (PRIME4DIA); Roche, Elecsys Anti-SARS-CoV-2 (Roche); SARS-CoV-2, severe acute respiratory syndrome coronavirus 2; SDF, STANDARD F COVID-19 IgM/IgG Combo FIA (SD BIOSENSOR); SDQ, STANDARD Q COVID-19 IgM/IgG Combo (SD BIOSENSOR); Siemens, ADVIA Centaur SARS-CoV-2 Total (Siemens).

### Kinetics of the Binding and Neutralizing Antibodies in Patients With Coronavirus Disease 2019

**FIGURE 2:**
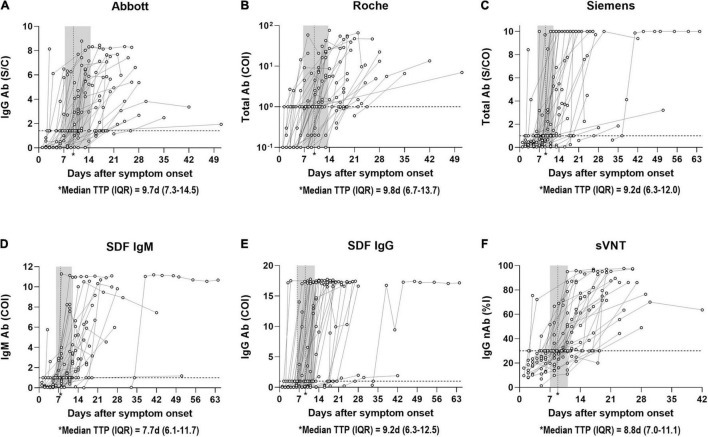
Distribution of time to seropositivity (TTP) of SARS-CoV-2 antibody immunoassays. **(A)** TTP of Abbott IgG antibody in 135 samples from 44 patients with PCR positive COVID-19 according to days post symptom onset. **(B)** TTP of Roche Total antibody in 121 samples from 41 patients with PCR positive COVID-19 according to days post symptom onset. **(C)** TTP of Siemens Total antibody in 132 samples from 39 patients with PCR positive COVID-19 according to days post symptom onset. **(D)** TTP of SDF IgM antibody in 125 samples from 37 patients with PCR positive COVID-19 according to days post symptom onset. **(E)** TTP of SDF IgG antibody in 139 samples from 42 patients with PCR positive COVID-19 according to days post symptom onset. **(F)** TTP of sVNT IgG neutralizing antibody in 133 samples from 41 patients with PCR positive COVID-19 according to days post symptom onset. The horizontal dotted line indicates the cut-off ratio for positivity. The vertical dotted line in the shaded area represents the median TTP with interquartile range. Each curve indicates the non-linear sigmoidal fit of circles of each patient. TTP is calculated by interpolating the positive cut-off line to the curve based on the four-parameter logistic (4PL) equation. Abbott, SARS-CoV-2 IgG (Abbott); Ab, antibody; COI, cut-off index; d, days; IQR, interquartile range; nAb, neutralizing antibody; %I, percentage inhibition; Roche, Elecsys Anti-SARS-CoV-2 (Roche); S/C, sample/calibrator; S/CO, sample/cut-off; SDF, STANDARD F COVID-19 IgM/IgG Combo FIA (SD BIOSENSOR); Siemens, ADVIA Centaur SARS-CoV-2 Total (Siemens); sVNT, surrogate virus neutralization test.

Kinetic analysis of symptomatic COVID-19 patients who demonstrated seroconversion based on the quantitative results of CLIA, SDF-IgM/IgG, and sVNT was performed (Figure 2). The seroconversion was detected in 135 serial samples from 44 patients by Abbott, 121 serial samples from 41 patients by Roche, 132 serial samples from 39 patients by Siemens, 125 serial samples from 37 patients by SDF-IgM, 139 serial samples from 42 patients by SDF-IgG, and 133 serial samples from 41 patients by sVNT. The distribution of time to seropositivity (TTP) was calculated by interpolating the positive cut-off line to the curve using the four-parameter logistic (4PL) equation (AAT Bioquest Inc, 2022). The median TTPs were as follows in the ascending order: 7.7 days for SDF-IgM, 8.8 days for sVNT, 9.2 days for Siemens and SDF-IgG, 9.7 days for Abbott, and 9.8 days for Roche. In addition, TTP for each assay was also analyzed in the asymptomatic group. The median TTPs were as follows in the ascending order: 7.2 days for sVNT (patient *n* = 3), 7.5 days for SDF-IgG (patient *n* = 3), 8.8 days for SDF-IgM (patient *n* = 2), 9.6 days for Roche (patient *n* = 4), 10.2 days for Siemens (patient *n* = 2), and 10.6 days for Abbott (patient *n* = 3).

### Correlation of Surrogate Virus Neutralization Test With Binding Antibody Assays

**FIGURE 3:**
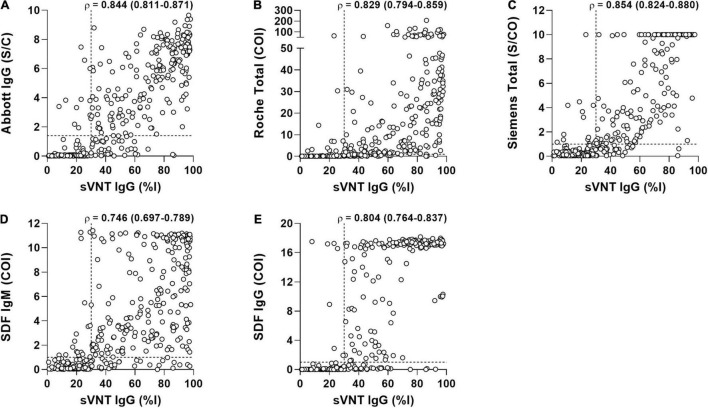
The correlation of surrogate virus neutralization test (sVNT) with the binding antibody assays in 389 samples from 199 COVID-19 patients. **(A)** Abbott vs. sVNT. **(B)** Roche vs. Siemens. **(C)** Siemens vs. sVNT. **(D)** SDF IgM vs. sVNT. **(E)** SDF IgG vs. sVNT. Each triangle represents an individual positive sample. Correlation between two measures was performed using Spearman ρ (95% confidence interval). The horizontal and vertical dotted lines indicate the cut-off value of each assay. Abbott, SARS-CoV-2 IgG (Abbott); COI, cut-off index; %I, percentage inhibition; Roche, Elecsys Anti-SARS-CoV-2 (Roche); S/C, sample/calibrator; S/CO, sample/cut-off; SDF, STANDARD F COVID-19 IgM/IgG Combo FIA (SD BIOSENSOR); Siemens, ADVIA Centaur SARS-CoV-2 Total (Siemens); sVNT, surrogate virus neutralization test.

The quantitative results of the three CLIAs, SDF-IgM/IgG, and sVNT acquired from COVID-19-positive samples were used to analyze the correlation between assays (Figure 3). The results showed a strong correlation between sVNT and other assays, with the Spearman ρ-values ranging from 0.746 (sVNT vs. SDF-IgM) to 0.854 (sVNT vs. Siemens).

## Discussion

To the best of our knowledge, this is the most extensive single-center evaluation of SARS-CoV-2 antibody tests in Korea, assessing the diagnostic performance of six different SARS-CoV-2 bAb assays and the activity and kinetics of neutralizing antibodies in a large set of COVID-19 samples.

Several SARS-CoV-2 antibody assays have been developed and evaluated. However, owing to the variable factors affecting diagnostic accuracy, the clinical implications remain uncertain. The reported performance of antibody assays varies widely by factors such as the size of patients or samples, the type of analytical method, the type of antigen, the population used as the control samples, or the timing of sample collection (Lisboa Bastos et al., 2020; Chvatal-Medina et al., 2021; Jarrom et al., 2022). Recently, a meta-analysis reported that the sensitivity of antibody tests using CLIAs ranged from 48.1 to 100%, and that of LFIAs ranged from 14.4 to 100% (Lisboa Bastos et al., 2020). In our study, the pooled sensitivity of CLIAs was 55.4%, and that of the LFIAs was 56.1%, with no significant difference between the two groups. Among the CLIAs, the Abbott was used to detect IgG, and there was no significant difference in the IgG detection rate between CLIAs and LFIAs. Among the LFIAs, the sensitivity of the combination of IgM and IgG was higher than that of each Ig class, consistent with findings from previous studies (Chen et al., 2020; Yun et al., 2021). Other studies have also recommended measuring both IgM and IgG in the first days of illness to reduce the risk of false-negative results, which may be due to dynamic antibody titer changes (Krajewski et al., 2020; Sun et al., 2020).

We analyzed the PPV, NPV, and accuracy with the rate of the accumulated confirmed cases (1.7%) in Korea. The PPV of Roche and Abbott was similar to the results of Park et al. (2022), who calculated the PPV using several exemplary COVID-19-prevalent populations. The PPV of Siemens was relatively lower than that of other CLIAs, with lower specificity compared to previous findings (Florin et al., 2021; Yun et al., 2021; Park et al., 2022). As the CLIAs for the SARS-CoV-2 antibody are not routinely used in our laboratory work, the reagent evaluation for this study was performed only for a short period. Therefore, insufficient optimization of the analytical system might be one reason for the poor performance (Kumleben et al., 2020; Ye et al., 2021).

Usually, the LFIA result appears in color-changing bands interpreted by visual inspection, which can easily be influenced by the observer’s experience and subjectivity. Those ambiguous bands would be a critical issue in using LFIAs for SARS-CoV-2 antibody detection because reclassifying trace bands as “negative” can change the test performance, as shown in our analysis (Table 4). A previous study underlined the importance of seropositive threshold determination, observer training, and LFIA analytical tools such as digital image analysis to improve objectivity (Whitman et al., 2020).

As reported in previous studies, our results showed that the antibody detection rate of the symptomatic group increased over 80% > 14 days after symptom onset in all assays (Sun et al., 2020; Nicholson et al., 2021; Yun et al., 2021). However, the rate in the asymptomatic group reached over 80% > 21 days after initial RT-PCR detection. This result might be due to the proportion difference in the early (1–7 days) stages of illness between the two groups. According to the Korean government’s rapid COVID-19 response system, the asymptomatic COVID-19 patients were generally confirmed through contact tracing; hence, the early stage proportion would be higher [Table 3: 30.5% (98/321) vs. 69.1% (47/68)]. Another explanation could be that a lower viral load in asymptomatic individuals leads to a lower seropositivity rate, as reported by Wellinghausen et al. (2020). Additionally, we identified the primary RT-PCR cycle threshold (Ct) values tested at CNUH for 148 of the 158 symptomatic patients and 34 of the 41 asymptomatic patients. The Ct values of both the envelope (*E*) and RNA-dependent RNA polymerase (*RdRp*) genes revealed approximately one cycle bias between the symptomatic and asymptomatic groups with no significant difference [*E* gene: mean ± SD, 24.44 ± 6.38 vs. 23.43 ± 5.64, *p* = 0.4064; *RdRp* gene: 25.33 ± 6.22 vs. 24.35 ± 5.38; *p* = 0.4495 (data not shown)]. However, since one cycle difference in PCR suggests twice the viral load, we could assume that the viral load of asymptomatic patients might be only about half of that of symptomatic patients in this study.

A previous study on false-positive results of SARS-CoV-2 antibody tests in samples stored before the pandemic reported that the false-positive rate of the LFIAs was higher than that of the ELISAs (1.8% vs. 0.6%) (Latiano et al., 2021). This was consistent with our results, in which the overall false-positive rates of the LFIAs were of a more expansive range than those of the CLIAs (Table 3). A large portion of the false-positive results was weak or trace, and the additionally performed sVNT was negative, except for in one sample collected in the prepandemic period. Among a total of 455 samples collected in the pandemic period, 9, 12, and 4 were found to be false positive for SDF-IgM/IgG, SDQ-IgM/IgG, and P4D-IgM/IgG, leading to assay specificities of 98.0, 97.4, and 99.1%, respectively. A comparative analysis for specificity, sensitivity, PPV, and NPV of all three LFIAs for varying seroprevalences (1, 5, and 10%) of SARS-CoV-2 antibody was shown in Supplementary Table 4. At seroprevalence of 10%, all LFIAs had unsatisfactory or acceptable PPVs of 78.3, 72.2, and 87.7% for SDF-IgM/IgG, SDQ-IgM/IgG, and P4D-IgM/IgG, but at seroprevalence of 1%, these values dropped to unacceptably low levels of 24.7, 19.1, and 39.3 for SDF, SDQ, and P4D. However, at varying seroprevalence of 1, 5, and 10%, NPVs ranging from 95.3 to 99.6% were acceptably high levels. Collectively, these findings suggest that LFIA tests may be useful in a high seroprevalence setting, in which COVID-19 is widely spread.

In SARS-CoV-2 antibody testing, false-positive results (non-specific or cross-reactive) can arise from endogenous factors such as rheumatoid factors, heterophil antibodies, lysozymes, complements, other cross-antigens (e.g., similar epitopes between SARS-CoV-2 and other human coronaviruses), or exogenous interferences such as inadequate specimen quality and unsatisfactory test kit optimization (Ye et al., 2021). A previous study in sub-Saharan Africa showed that pre-pandemic plasma samples, which either had the S proteins of HCoV-OC43, HCoV-HKU-1, HCoV-NL63, and HCoV-229E or the N proteins of HCoV-NL63, and HCoV-229E, were serological cross-reactive against the S and N proteins of SARS-CoV-2 (Tso et al., 2021). Previous studies have also reported cross-reactivity with autoantibodies such as ANA and other viral infections such as cytomegalovirus (Jääskeläinen et al., 2020; Nicholson et al., 2021). In practice, false-positive cases are difficult to rule out; therefore, test subjects should be selected wisely, recognizing the limitations of serological tests when applying them to asymptomatic, healthy subjects with no history of SARS-CoV-2 exposure (Latiano et al., 2021).

In the agreement rate analysis, all assays showed good agreement. Among the CLIAs, the Abbott vs. Roche comparison had a higher agreement rate than the Siemens vs. Abbott or Roche comparison, consistent with other studies (Yun et al., 2021; Park et al., 2022). This result might be due to the difference in the target protein—Abbott and Roche target an epitope of the N protein, and Siemens targets the S protein. Among the LFIAs, SDF-IgG and SDQ-IgG showed the highest agreement rates, likely because both assays are from the same manufacturer and target the same IgG.

The sVNT test indirectly detects the function of neutralizing SARS-CoV-2 antibodies that block the interaction between the viral spike RBD and the host ACE2 receptor. The positive rate of sVNT in sera collected > 14 days after symptom onset was similar to previous findings (Tan et al., 2020; Nicholson et al., 2021; Yun et al., 2021). Interestingly, the symptomatic group showed higher inhibition activity than the asymptomatic group, although the positive rate was reversed (Table 3 and Figure 1F). These data might align with the statement that the asymptomatic group consisted of a higher proportion of early stage illnesses and might have a lower viral load (Wellinghausen et al., 2020). However, this could be due to the sample size imbalance (8 vs. 112) between asymptomatic and symptomatic groups at that time frame (≥15 days category), requiring a further study using more sample size of asymptomatic cases. In our study, the bAb IgM assay showed the earliest seroconversion at 7.7 days, followed by the nAb IgG assay at 8.8 days and the bAb IgG assay at 9.2–9.8 days, which supports previous data for the utility and clinical importance of using IgM antibodies for SARS-CoV-2 diagnosis (Ng et al., 2020; Orner et al., 2021). The index values of the three CLIAs and SDF-IgM/IgG vs. sVNT percentage inhibition were strongly correlated (Figure 3), and the use of the same target protein could explain the strongest correlation between Siemens and sVNT (Yun et al., 2021).

Our study had some limitations. First, the negative samples were from patients in the pre-pandemic and pandemic periods who had no history of COVID-19 or epidemiological relationship with COVID-19. They did not undergo additional PCR confirmation. Nevertheless, reflecting the government’s strict response policy to COVID-19 and the low disease prevalence of COVID-19 at that time in Korea, the samples from patients with no history of COVID-19 or any epidemiological relationship with COVID-19 could be considered COVID-19-negative. Second, false-negative (or even undetected) results cannot be ruled out due to the possibility of insufficient optimization of the assay systems, as we evaluated the assays only for a short period. Finally, we only proposed the fragmentary kinetics of the antibodies detected in this study. Because the samples used in this study were serum remnants from blood samples retrieved for routine laboratory tests, the multiple samples from one patient were serially collected at different time points (one or two samples per week) but having irregular time intervals, providing only estimated TTPs for individual patients calculated by interpolating the positive cut-off line to the curve using the 4PL equation. Moreover, the SARS-CoV-2 antibody response is correlated with various factors, including primary infection or reinfection of COVID-19, symptom onset, disease severity, fever, age, and sex (Schlickeiser et al., 2021). As the national contact tracing system was widely and strictly applied to all patients from the beginning of the pandemic period in Korea, the contact history with COVID-19 patients in this study were thoroughly investigated through the contact tracing system. As a result of the analysis, all of these patients had a current but no previous contact history with COVID-19 patients in the pandemic period, indicating that all of our patients had a primary infection, not re-infection. In addition, we analyzed the difference of antibody kinetics between symptomatic and asymptomatic groups. However, the sample size of the asymptomatic group was too small to calculate the *p*-values. More serial follow-up data and large-sized samples from well-evaluated individuals may be needed for intense antibody kinetic analyses.

In conclusion, our study offers a detailed comparison of three CLIAs, three LFIAs, and an sVNT assay. With the initiation of vaccines administration, routine antibody test for COVID-19 has been started in general laboratories all over the world. Therefore, to choose the most suitable serological assays for a particular laboratory environment and situation, it is necessary to understand the characteristics of each assay. The interpretation of antibody assay results should also be performed with caution. The patient’s contact history, symptoms, the time of illness, measured assays, target antibodies, and the antigens used should also be considered. In particular, for LFIAs, it is recommended that more objectively interpreted assays are used, and a band interpretation system should be established for each laboratory with sufficient observer training. We also expect that routinely available sVNT will play an essential role in the laboratory where nAb testing is desired.

## Data Availability Statement

The original contributions presented in the study are included in the article/Supplementary Material, further inquiries can be directed to the corresponding author/s.

## Ethics Statement

The studies involving human participants were reviewed and approved by the Institutional Review Board (IRB) of CNUH (IRB No. CNUH-2020-223). Written informed consent for participation was not required for this study in accordance with the national legislation and the institutional requirements.

## Author Contributions

H-WC, SYL, and S-JKe designed the study, analyzed, and discussed the data. H-WC, C-HJ, S-JKa, EJW, and SYL generated the data. H-WC and SYL ran the statistical analysis. H-WC and S-JKe wrote the primary manuscript. All authors were involved in writing and had final approval of the submitted and published versions of the manuscript.

## Conflict of Interest

The authors declare that the research was conducted in the absence of any commercial or financial relationships that could be construed as a potential conflict of interest.

## Publisher’s Note

All claims expressed in this article are solely those of the authors and do not necessarily represent those of their affiliated organizations, or those of the publisher, the editors and the reviewers. Any product that may be evaluated in this article, or claim that may be made by its manufacturer, is not guaranteed or endorsed by the publisher.

## Footnotes

1
www.medcalc.org
