# Supplementary material for: Performance of Severe Acute Respiratory Syndrome Coronavirus 2 Serological Diagnostic Tests and Antibody Kinetics in Coronavirus Disease 2019 Patients
Source: Front Microbiol. 2022 Apr 14;13:881038. doi: 10.3389/fmicb.2022.881038 (PMC9048255; doi:10.3389/fmicb.2022.881038)
Supplement: Supplementary file 1 [file Table_1.docx]

**Supplementary Table 1.** Sampling days of multiple samples per patient for SARS-CoV-2 antibody assays in this study

| **PID** | **Sampling days after initial RT-PCR (+)** | | | | | | | | | **No. serum  per patient** | **No.  patients** |
| --- | --- | --- | --- | --- | --- | --- | --- | --- | --- | --- | --- |
|  | **1st** | **2nd** | **3rd** | **4th** | **5th** | **6th** | **7th** | **8th** | **9th** |  |  |
| 1 | 0 | 7 |  |  |  |  |  |  |  | 2 | 48 |
| 2 | 8 | 11 |  |  |  |  |  |  |  | 2 |  |
| 3 | 10 | 23 |  |  |  |  |  |  |  | 2 |  |
| 4 | 14 | 34 |  |  |  |  |  |  |  | 2 |  |
| 5 | 9 | 15 |  |  |  |  |  |  |  | 2 |  |
| 6 | 1 | 4 |  |  |  |  |  |  |  | 2 |  |
| 7 | 15 | 25 |  |  |  |  |  |  |  | 2 |  |
| 8 | 1 | 5 |  |  |  |  |  |  |  | 2 |  |
| 9 | 42 | 58 |  |  |  |  |  |  |  | 2 |  |
| 10 | 4 | 12 |  |  |  |  |  |  |  | 2 |  |
| 11 | 1 | 8 |  |  |  |  |  |  |  | 2 |  |
| 12 | 6 | 12 |  |  |  |  |  |  |  | 2 |  |
| 13 | 1 | 2 |  |  |  |  |  |  |  | 2 |  |
| 14 | 1 | 6 |  |  |  |  |  |  |  | 2 |  |
| 15 | 8 | 9 |  |  |  |  |  |  |  | 2 |  |
| 16 | 3 | 9 |  |  |  |  |  |  |  | 2 |  |
| 17 | 1 | 3 |  |  |  |  |  |  |  | 2 |  |
| 18 | 3 | 9 |  |  |  |  |  |  |  | 2 |  |
| 19 | 7 | 23 |  |  |  |  |  |  |  | 2 |  |
| 20 | 1 | 48 |  |  |  |  |  |  |  | 2 |  |
| 21 | 1 | 6 |  |  |  |  |  |  |  | 2 |  |
| 22 | 1 | 30 |  |  |  |  |  |  |  | 2 |  |
| 23 | 2 | 7 |  |  |  |  |  |  |  | 2 |  |
| 24 | 12 | 15 |  |  |  |  |  |  |  | 2 |  |
| 25 | 2 | 6 |  |  |  |  |  |  |  | 2 |  |
| 26 | 5 | 8 |  |  |  |  |  |  |  | 2 |  |
| 27 | 2 | 9 |  |  |  |  |  |  |  | 2 |  |
| 28 | 6 | 12 |  |  |  |  |  |  |  | 2 |  |
| 29 | 1 | 5 |  |  |  |  |  |  |  | 2 |  |
| 30 | 1 | 7 |  |  |  |  |  |  |  | 2 |  |
| 31 | 7 | 10 |  |  |  |  |  |  |  | 2 |  |
| 32 | 10 | 18 |  |  |  |  |  |  |  | 2 |  |
| 33 | 2 | 14 |  |  |  |  |  |  |  | 2 |  |
| 34 | 10 | 16 |  |  |  |  |  |  |  | 2 |  |
| 35 | 9 | 9 |  |  |  |  |  |  |  | 2 |  |
| 36 | 1 | 16 |  |  |  |  |  |  |  | 2 |  |
| 37 | 6 | 30 |  |  |  |  |  |  |  | 2 |  |
| 38 | 7 | 15 |  |  |  |  |  |  |  | 2 |  |
| 39 | 2 | 10 |  |  |  |  |  |  |  | 2 |  |
| 40 | 15 | 26 |  |  |  |  |  |  |  | 2 |  |
| 41 | 11 | 15 |  |  |  |  |  |  |  | 2 |  |
| 42 | 19 | 33 |  |  |  |  |  |  |  | 2 |  |
| 43 | 10 | 16 |  |  |  |  |  |  |  | 2 |  |
| 44 | 11 | 17 |  |  |  |  |  |  |  | 2 |  |
| 45 | 7 | 11 |  |  |  |  |  |  |  | 2 |  |
| 46 | 20 | 41 |  |  |  |  |  |  |  | 2 |  |
| 47 | 2 | 10 |  |  |  |  |  |  |  | 2 |  |
| 48 | 1 | 4 |  |  |  |  |  |  |  | 2 |  |
| 49 | 1 | 9 | 25 |  |  |  |  |  |  | 3 | 27 |
| 50 | 10 | 15 | 18 |  |  |  |  |  |  | 3 |  |
| 51 | 3 | 8 | 20 |  |  |  |  |  |  | 3 |  |
| 52 | 2 | 7 | 10 |  |  |  |  |  |  | 3 |  |
| 53 | 2 | 5 | 8 |  |  |  |  |  |  | 3 |  |
| 54 | 4 | 8 | 9 |  |  |  |  |  |  | 3 |  |
| 55 | 1 | 6 | 9 |  |  |  |  |  |  | 3 |  |
| 56 | 1 | 5 | 7 |  |  |  |  |  |  | 3 |  |
| 57 | 2 | 5 | 8 |  |  |  |  |  |  | 3 |  |
| 58 | 3 | 10 | 17 |  |  |  |  |  |  | 3 |  |
| 59 | 1 | 6 | 7 |  |  |  |  |  |  | 3 |  |
| 60 | 4 | 7 | 10 |  |  |  |  |  |  | 3 |  |
| 61 | 11 | 13 | 21 |  |  |  |  |  |  | 3 |  |
| 62 | 3 | 9 | 15 |  |  |  |  |  |  | 3 |  |
| 63 | 2 | 5 | 15 |  |  |  |  |  |  | 3 |  |
| 64 | 6 | 12 | 19 |  |  |  |  |  |  | 3 |  |
| 65 | 11 | 18 | 23 |  |  |  |  |  |  | 3 |  |
| 66 | 8 | 10 | 14 |  |  |  |  |  |  | 3 |  |
| 67 | 1 | 3 | 9 |  |  |  |  |  |  | 3 |  |
| 68 | 0 | 9 | 27 |  |  |  |  |  |  | 3 |  |
| 69 | 8 | 16 | 23 |  |  |  |  |  |  | 3 |  |
| 70 | 1 | 4 | 12 |  |  |  |  |  |  | 3 |  |
| 71 | 6 | 12 | 18 |  |  |  |  |  |  | 3 |  |
| 72 | 1 | 4 | 10 |  |  |  |  |  |  | 3 |  |
| 73 | 1 | 5 | 9 |  |  |  |  |  |  | 3 |  |
| 74 | 4 | 9 | 16 |  |  |  |  |  |  | 3 |  |
| 75 | 0 | 9 | 10 |  |  |  |  |  |  | 3 |  |
| 76 | 3 | 7 | 10 | 13 |  |  |  |  |  | 4 | 12 |
| 77 | 2 | 5 | 10 | 14 |  |  |  |  |  | 4 |  |
| 78 | 2 | 5 | 15 | 19 |  |  |  |  |  | 4 |  |
| 79 | 1 | 5 | 9 | 28 |  |  |  |  |  | 4 |  |
| 80 | 4 | 7 | 17 | 31 |  |  |  |  |  | 4 |  |
| 81 | 1 | 6 | 10 | 14 |  |  |  |  |  | 4 |  |
| 82 | 2 | 9 | 14 | 23 |  |  |  |  |  | 4 |  |
| 83 | 4 | 7 | 7 | 10 |  |  |  |  |  | 4 |  |
| 84 | 7 | 12 | 20 | 25 |  |  |  |  |  | 4 |  |
| 85 | 3 | 10 | 17 | 24 |  |  |  |  |  | 4 |  |
| 86 | 1 | 4 | 7 | 23 |  |  |  |  |  | 4 |  |
| 87 | 2 | 6 | 9 | 21 |  |  |  |  |  | 4 |  |
| 88 | 4 | 10 | 14 | 17 | 19 |  |  |  |  | 5 | 6 |
| 89 | 1 | 5 | 10 | 14 | 21 |  |  |  |  | 5 |  |
| 90 | 1 | 11 | 18 | 25 | 37 |  |  |  |  | 5 |  |
| 91 | 1 | 5 | 10 | 17 | 18 |  |  |  |  | 5 |  |
| 92 | 1 | 7 | 14 | 21 | 42 |  |  |  |  | 5 |  |
| 93 | 0 | 7 | 12 | 16 | 21 |  |  |  |  | 5 |  |
| 94 | 2 | 6 | 11 | 12 | 15 | 16 |  |  |  | 6 | 1 |
| 95 | 8 | 10 | 12 | 13 | 14 | 15 | 16 | 17 |  | 8 | 1 |
| 96 | 1 | 6 | 9 | 12 | 16 | 18 | 23 | 28 | 32 | 9 | 2 |
| 97 | 1 | 6 | 7 | 12 | 14 | 16 | 19 | 23 | 25 | 9 |  |
| **Median** | **2.0** | **9.0** | **12.0** | **17.0** | **18.5** | **16.0** | **19.0** | **23.0** | **28.5** | **3.0** |  |
| **25th** | **1.0** | **6.0** | **9.0** | **13.0** | **14.8** | **15.3** | **16.0** | **17.0** |  | **2.0** |  |
| **75th** | **7.0** | **13.5** | **17.0** | **23.3** | **25.0** | **17.5** | **23.0** | **28.0** |  | **3.0** |  |

*Abbreviation*: No. number; PID, patient identification; 25th, 25th percentile; 75th, 75th percentile.
