# Supplementary material for: Performance of Severe Acute Respiratory Syndrome Coronavirus 2 Serological Diagnostic Tests and Antibody Kinetics in Coronavirus Disease 2019 Patients
Source: Front Microbiol. 2022 Apr 14;13:881038. doi: 10.3389/fmicb.2022.881038 (PMC9048255; doi:10.3389/fmicb.2022.881038)
Supplement: Supplementary file 2 [file Table_2.docx]

**Supplementary Table 2.** False-positive results of SARS-CoV-2 antibody assays in known negative samples

| \| **Discordance number for** \| \| **Case ID** \| **Abbott** \|  \| **Roche** \|  \| **Siemens** \|  \| **SDF** \| \|  \| **SDQ** \| \|  \| **P4D** \| \|  \| **sVNT** \| **Note** \| \| --- \| --- \| --- \| --- \| --- \| --- \| --- \| --- \| --- \| --- \| --- \| --- \| --- \| --- \| --- \| --- \| --- \| --- \| --- \| --- \| \| **Assay** \| **Sample** \| **IgG (S/C)** \|  \| **Total (COI)** \|  \| **Total (S/CO)** \|  \| **IgM (COI)** \| **IgG (COI)** \|  \| **IgM** \| **IgG** \|  \| **IgM** \| **IgG** \|  \| **IgG (%)** \| \| 1 \| 20 \| 110 \| N \|  \| N \|  \| N \|  \| N \| N \|  \| P (trace) \| N \|  \| N \| N \|  \| NT \| ANA (+), pre-pandemic \| \|  \|  \| 117 \| N \|  \| N \|  \| N \|  \| N \| N \|  \| P (trace) \| N \|  \| N \| N \|  \| NT \| ANA (+), pre-pandemic \| \|  \|  \| 135 \| N \|  \| N \|  \| P (2.17) \|  \| N \| N \|  \| N \| N \|  \| N \| N \|  \| N (7.3) \| HBV Ab (+) \| \|  \|  \| 138 \| N \|  \| N \|  \| N \|  \| N \| P (1.99) \|  \| N \| N \|  \| N \| N \|  \| N (9.8) \| HBV Ab (+) \| \|  \|  \| 157 \| N \|  \| N \|  \| P (2.14) \|  \| N \| N \|  \| N \| N \|  \| N \| N \|  \| N (17.2) \| HBV \| \|  \|  \| 342 \| N \|  \| N \|  \| P (1.97) \|  \| N \| N \|  \| N \| N \|  \| N \| N \|  \| N (7.7) \| Syphilis \| \|  \|  \| 352 \| N \|  \| N \|  \| P (1.01) \|  \| N \| N \|  \| N \| N \|  \| N \| N \|  \| N (10.0) \| HCV, HBV Ab (+) \| \|  \|  \| 360 \| N \|  \| N \|  \| P (7.24) \|  \| N \| N \|  \| N \| N \|  \| N \| N \|  \| N (18.6) \| HCV \| \|  \|  \| 362 \| P (1.68) \|  \| N \|  \| N \|  \| N \| N \|  \| N \| N \|  \| N \| N \|  \| N (22.4) \| HCV \| \|  \|  \| 381 \| N \|  \| N \|  \| N \|  \| P (1.07) \| N \|  \| N \| N \|  \| N \| N \|  \| N (22.4) \| HCV, HBV Ab (+) \| \|  \|  \| 388 \| N \|  \| N \|  \| P (7.20) \|  \| N \| N \|  \| N \| N \|  \| N \| N \|  \| N (24.3) \| CMV IgG/IgM (+) \| \|  \|  \| 407 \| N \|  \| P (1.54) \|  \| N \|  \| N \| N \|  \| N \| N \|  \| N \| N \|  \| N (11.9) \| HBV Ab (+) \| \|  \|  \| 501 \| N \|  \| N \|  \| N \|  \| N \| P (17.80) \|  \| N \| N \|  \| N \| N \|  \| P (34.0) \| ANA (+), pre-pandemic \| \|  \|  \| 520 \| N \|  \| N \|  \| N \|  \| N \| P (17.22) \|  \| N \| N \|  \| N \| N \|  \| N (1.9) \| ANA (+), pre-pandemic \| \|  \|  \| 554 \| N \|  \| N \|  \| P (>10.00) \|  \| N \| N \|  \| N \| N \|  \| N \| N \|  \| N (13.8) \| HAV IgM (+) \| \|  \|  \| 564 \| N \|  \| N \|  \| N \|  \| N \| N \|  \| N \| N \|  \| N \| P \|  \| NT \| HBV Ab (+) \| \|  \|  \| 565 \| N \|  \| N \|  \| P (1.51) \|  \| N \| N \|  \| N \| N \|  \| N \| N \|  \| N (19.8) \| HBV Ab (+) \| \|  \|  \| 811 \| N \|  \| N \|  \| P (1.92) \|  \| N \| N \|  \| N \| N \|  \| N \| N \|  \| NT \| HBV \| \|  \|  \| 947 \| P (1.68) \|  \| N \|  \| N \|  \| N \| N \|  \| N \| N \|  \| N \| N \|  \| N (19.8) \| ANA (+) \| \|  \|  \| 976 \| N \|  \| N \|  \| N \|  \| N \| N \|  \| P (trace) \| N \|  \| N \| N \|  \| N (15.8) \| Tsutsugamushi Ab (+) \| \| 2 \| 11 \| 136 \| N \|  \| N \|  \| N \|  \| P (2.10) \| N \|  \| P (trace) \| N \|  \| N \| N \|  \| N (7.5) \| HBV Ab (+) \| \|  \|  \| 145 \| N \|  \| N \|  \| N \|  \| N \| P (8.57) \|  \| N \| P (trace) \|  \| N \| N \|  \| N (15.7) \| HAV Ab (+), HBV Ab (+) \| \|  \|  \| 356 \| N \|  \| N \|  \| N \|  \| N \| N \|  \| P \| N \|  \| P (trace) \| N \|  \| N (8.6) \| CMV IgG (+) \| \|  \|  \| 394 \| N \|  \| N \|  \| P (1.96) \|  \| N \| N \|  \| P (trace) \| N \|  \| N \| N \|  \| N (21.5) \| Tsutsugamushi Ab (+) \| \|  \|  \| 396 \| P (1.80) \|  \| N \|  \| N \|  \| N \| N \|  \| P \| N \|  \| N \| N \|  \| N (20.9) \| Tsutsugamushi Ab (+) \| \|  \|  \| 608 \| N \|  \| N \|  \| N \|  \| N \| N \|  \| P (trace) \| N \|  \| P (trace) \| N \|  \| NT \| HBV \| \|  \|  \| 706 \| N \|  \| N \|  \| P (1.33) \|  \| P (1.22) \| N \|  \| N \| N \|  \| N \| N \|  \| N (21.9) \| ANA (+), pre-pandemic \| \|  \|  \| 903 \| N \|  \| N \|  \| N \|  \| P (1.54) \| N \|  \| P (trace) \| N \|  \| N \| N \|  \| N (13.9) \| ANA (+) \| \|  \|  \| 917 \| N \|  \| N \|  \| N \|  \| N \| P (7.27) \|  \| N \| P (trace) \|  \| N \| N \|  \| N (17.2) \| ANA (+) \| \|  \|  \| 989 \| N \|  \| N \|  \| N \|  \| P (1.14) \| N \|  \| P (trace) \| N \|  \| N \| N \|  \| N (13.1) \| HBV Ab (+) \| \|  \|  \| 1012 \| N \|  \| N \|  \| N \|  \| P (2.05) \| N \|  \| P (trace) \| N \|  \| N \| N \|  \| N (23.2) \| HBV Ab (+) \| \| 3 \| 3 \| 320 \| N \|  \| N \|  \| N \|  \| P (7.42) \| N \|  \| P (trace) \| N \|  \| P (trace) \| N \|  \| N (17.4) \| ANA (+), pre-pandemic \| \|  \|  \| 507 \| N \|  \| N \|  \| N \|  \| P (9.22) \| N \|  \| P (trace) \| N \|  \| P (trace) \| N \|  \| N (1.5) \| ANA (+), pre-pandemic \| \|  \|  \| 605 \| N \|  \| N \|  \| N \|  \| P (1.06) \| N \|  \| P (trace) \| N \|  \| P (trace) \| N \|  \| N (23.9) \| HBV Ab (+) \|   *Abbreviation*: Ab, antibody; Abbott, SARS-CoV-2 IgG (Abbott); ANA, antinuclear antibody; CMV, cytomegalovirus; COI, cut-off index; COVID-19, coronavirus disease 2019; HBV, hepatitis B virus; HCV, hepatitis C virus; ID, identification; N, negative; NT, not tested; P4D, P4DETECT COVID-19 IgM/IgG (PRIME4DIA); P, positive; Roche, Elecsys Anti-SARS-CoV-2 (Roche); SDF, STANDARD F COVID-19 IgM/IgG Combo FIA (SD BIOSENSOR); SDQ, STANDARD Q COVID-19 IgM/IgG Combo (SD BIOSENSOR); SARS-CoV-2, severe acute respiratory syndrome coronavirus 2; S/C, sample/calibrator; S/CO, sample/cut-off; Siemens, ADVIA Centaur SARS-CoV-2 Total (Siemens). |
| --- | --- | --- | --- | --- | --- | --- | --- | --- | --- | --- | --- | --- | --- | --- | --- | --- | --- | --- | --- | --- | --- | --- | --- | --- | --- | --- | --- | --- | --- | --- | --- | --- | --- | --- | --- | --- | --- | --- | --- | --- | --- | --- | --- | --- | --- | --- | --- | --- | --- | --- | --- | --- | --- | --- | --- | --- | --- | --- | --- | --- | --- | --- | --- | --- | --- | --- | --- | --- | --- | --- | --- | --- | --- | --- | --- | --- | --- | --- | --- | --- | --- | --- | --- | --- | --- | --- | --- | --- | --- | --- | --- | --- | --- | --- | --- | --- | --- | --- | --- | --- | --- | --- | --- | --- | --- | --- | --- | --- | --- | --- | --- | --- | --- | --- | --- | --- | --- | --- | --- | --- | --- | --- | --- | --- | --- | --- | --- | --- | --- | --- | --- | --- | --- | --- | --- | --- | --- | --- | --- | --- | --- | --- | --- | --- | --- | --- | --- | --- | --- | --- | --- | --- | --- | --- | --- | --- | --- | --- | --- | --- | --- | --- | --- | --- | --- | --- | --- | --- | --- | --- | --- | --- | --- | --- | --- | --- | --- | --- | --- | --- | --- | --- | --- | --- | --- | --- | --- | --- | --- | --- | --- | --- | --- | --- | --- | --- | --- | --- | --- | --- | --- | --- | --- | --- | --- | --- | --- | --- | --- | --- | --- | --- | --- | --- | --- | --- | --- | --- | --- | --- | --- | --- | --- | --- | --- | --- | --- | --- | --- | --- | --- | --- | --- | --- | --- | --- | --- | --- | --- | --- | --- | --- | --- | --- | --- | --- | --- | --- | --- | --- | --- | --- | --- | --- | --- | --- | --- | --- | --- | --- | --- | --- | --- | --- | --- | --- | --- | --- | --- | --- | --- | --- | --- | --- | --- | --- | --- | --- | --- | --- | --- | --- | --- | --- | --- | --- | --- | --- | --- | --- | --- | --- | --- | --- | --- | --- | --- | --- | --- | --- | --- | --- | --- | --- | --- | --- | --- | --- | --- | --- | --- | --- | --- | --- | --- | --- | --- | --- | --- | --- | --- | --- | --- | --- | --- | --- | --- | --- | --- | --- | --- | --- | --- | --- | --- | --- | --- | --- | --- | --- | --- | --- | --- | --- | --- | --- | --- | --- | --- | --- | --- | --- | --- | --- | --- | --- | --- | --- | --- | --- | --- | --- | --- | --- | --- | --- | --- | --- | --- | --- | --- | --- | --- | --- | --- | --- | --- | --- | --- | --- | --- | --- | --- | --- | --- | --- | --- | --- | --- | --- | --- | --- | --- | --- | --- | --- | --- | --- | --- | --- | --- | --- | --- | --- | --- | --- | --- | --- | --- | --- | --- | --- | --- | --- | --- | --- | --- | --- | --- | --- | --- | --- | --- | --- | --- | --- | --- | --- | --- | --- | --- | --- | --- | --- | --- | --- | --- | --- | --- | --- | --- | --- | --- | --- | --- | --- | --- | --- | --- | --- | --- | --- | --- | --- | --- | --- | --- | --- | --- | --- | --- | --- | --- | --- | --- | --- | --- | --- | --- | --- | --- | --- | --- | --- | --- | --- | --- | --- | --- | --- | --- | --- | --- | --- | --- | --- | --- | --- | --- | --- | --- | --- | --- | --- | --- | --- | --- | --- | --- | --- | --- | --- | --- | --- | --- | --- | --- | --- | --- | --- | --- | --- | --- | --- | --- | --- | --- | --- | --- | --- | --- | --- | --- | --- | --- | --- | --- | --- | --- | --- | --- | --- | --- | --- | --- | --- | --- | --- | --- | --- | --- | --- | --- | --- | --- | --- | --- | --- | --- | --- | --- | --- | --- | --- | --- | --- | --- | --- | --- | --- | --- | --- | --- | --- | --- | --- | --- | --- | --- | --- | --- | --- | --- | --- | --- | --- | --- | --- | --- | --- | --- | --- | --- | --- | --- | --- | --- | --- | --- | --- | --- | --- | --- | --- | --- | --- | --- | --- | --- | --- | --- | --- | --- | --- | --- | --- | --- | --- | --- | --- | --- | --- | --- | --- | --- | --- | --- | --- | --- | --- | --- | --- | --- | --- | --- | --- | --- | --- | --- | --- | --- | --- | --- | --- | --- | --- | --- | --- | --- | --- | --- | --- | --- | --- | --- | --- | --- | --- | --- | --- | --- | --- | --- | --- | --- | --- | --- | --- | --- | --- | --- | --- | --- | --- | --- | --- | --- | --- | --- | --- | --- | --- | --- | --- | --- | --- | --- | --- | --- | --- | --- | --- | --- | --- | --- | --- | --- | --- | --- | --- | --- | --- | --- | --- | --- | --- | --- | --- | --- | --- | --- | --- | --- | --- | --- | --- | --- | --- | --- | --- | --- | --- | --- | --- | --- | --- | --- | --- |
