# Supplementary material for: Performance of Severe Acute Respiratory Syndrome Coronavirus 2 Serological Diagnostic Tests and Antibody Kinetics in Coronavirus Disease 2019 Patients
Source: Front Microbiol. 2022 Apr 14;13:881038. doi: 10.3389/fmicb.2022.881038 (PMC9048255; doi:10.3389/fmicb.2022.881038)
Supplement: Supplementary file 3 [file Table_3.DOCX]

**Supplementary Table 3.** Positive and negative percent agreement between SARS-CoV-2 antibody assays used in this study

| % (95% CI)  NPA  PPA  % (95% CI) | Abbott-  IgG | Roche-  Total | Siemens-Total | SDF-  IgM | SDF-  IgG | SDF-  IgM/IgG | SDQ-  IgM | SDQ-  IgG | SDQ-  IgM/IgG | P4D-  IgM | P4D-  IgG | P4D-  IgM/IgG |
| --- | --- | --- | --- | --- | --- | --- | --- | --- | --- | --- | --- | --- |
| Abbott-IgG |  | 98.7  (97.6−99.4) | 95.3  (93.6−96.7) | 94.4  (92.6−95.9) | 97.7  (96.3−98.6) | 93.1  (91.1−94.8) | 95.1  (93.3−96.5) | 98.2  (97.0−99.0) | 94.2  (92.3−95.7) | 96.6  (95.1−97.8) | 98.7  (97.6−99.4) | 95.7  (94.3−97.3) |
| Roche-Total | 92.7 (88.4−95.8) |  | 95.1  (93.3−96.5) | 93.6  (91.6−95.2) | 97.0  (95.6−98.1) | 92.4  (90.3−94.2) | 94.3  (92.5−95.9) | 97.4  (96.1−98.4) | 93.4  (91.5−95.1) | 96.1  (94.5−97.4) | 98.2  (97.0−99.0) | 95.5  (93.8−96.8 |
| Siemens-Total | 89.5  (84.6−93.2) | 91.0  (86.4−94.5) |  | 94.3  (92.4−95.9) | 97.0  (95.5−98.1) | 93.1  (91.1−94.8) | 95.0  (93.2−96.4) | 97.2  (95.8−98.3) | 93.9  (92.0−95.5) | 96.8  (95.3−98.0) | 98.3  (97.1−99.1) | 95.9  (94.2−97.2) |
| SDF-IgM | 89.5  (84.6−93.2) | 88.7  (83.6−92.6) | 84.2  (78.8−88.7) |  | 96.5  (95.0−97.7) | 96.5  (95.0−97.7) | 98.3  (97.1−99.1) | 96.3  (94.7−97.5) | 95.2  (93.4−96.6) | 99.2  (98.3−99.7) | 97.5  (96.1−98.5) | 97.2  (95.8−98.3) |
| SDF-IgG | 91.7  (87.3−95.0) | 92.0  (87.5−95.3) | 84.4  (79.1−88.8) | 80.7  (75.1−85.5) |  | 94.0  (92.1−95.6) | 94.8  (93.0−96.3) | 98.7  (97.6−99.4) | 94.2  (92.3−95.7) | 96.6  (95.1−97.8) | 99.6  (98.9−99.9) | 96.4  (94.8−97.6) |
| SDF-IgM/IgG | 96.8  (93.5−98.7) | 96.7  (93.3−98.7) | 91.8  (87.5−95.0) | 100  (98.5−100) | 100  (98.3−100) |  | 98.8  (97.7−99.4) | 99.2  (98.2−99.7) | 98.1  (96.8−98.9) | 99.6  (98.8−99.9) | 99.6  (98.9−99.9) | 99.3  (98.4−99.8) |
| SDQ-IgM | 89.0  (84.1−92.8) | 88.7  (83.6−92.6) | 84.0  (78.6−88.5) | 92.0  (87.8−95.1) | 88.1  (83.0−92.1) | 84.5  (76.5−88.6) |  | 96.8  (95.3−98.0) | 96.8  (95.3−98.0) | 100  (99.5−100) | 98.0  (96.8−98.9) | 98.0  (96.8−98.9) |
| SDQ-IgG | 92.7  (88.4−95.8) | 92.5  (88.0−95.6) | 84.4  (79.1−88.8) | 79.0  (73.3−84.0) | 94.5  (90.6−97.1) | 79.6  (74.2−84.2) | 82.8  (77.3−87.4) |  | 94.8  (93.0−96.3) | 96.4  (94.8−97.6) | 99.5  (98.7−99.9) | 96.1  (94.5−97.4) |
| SDQ-IgM/IgG | 96.8  (93.5−98.7) | 96.7  (93.3−98.7) | 90.9  (86.4−94.3) | 92.4  (88.3−95.5) | 96.8  (93.5−98.7) | 91.7  (87.7−94.7) | 100  (98.4−100) | 100  (98.3−100) |  | 100  (99.5−100) | 99.7  (99.0−100) | 99.7  (99.0−100) |
| P4D-IgM | 79.4  (73.4−84.5) | 79.7  (73.7−84.9) | 75.8  (69.7−81.1) | 81.1  (75.5−85.9) | 79.4  (73.4−84.5) | 74.2  (68.5−79.4) | 85.8  (80.6−90.0) | 79.2  (73.1−84.4) | 77.7  (72.1−82.7) |  | 96.7  (95.2−97.8) | 96.7  (95.2−97.8) |
| P4D-IgG | 81.2  (75.4−86.2) | 81.6  (75.7−86.6) | 75.3  (69.2−80.7) | 70.6  (64.4−76.3) | 84.4  (78.9−89.0) | 69.7  (63.8−75.2) | 74.1  (68.0−79.7) | 84.7  (79.2−89.2) | 72.3  (66.4−77.7) | 80.9  (74.8−86.1) |  | 95.3  (93.6−96.6) |
| P4D-IgM/IgG | 89.0  (84.1−92.8) | 89.6  (84.7−93.4) | 84.0  (78.6−88.5) | 85.7  (80.6−89.9) | 90.4  (85.7−93.9) | 83.3  (78.3−87.6) | 90.5  (86.0−94.0) | 90.3  (85.5−93.9) | 87.1  (82.4−91.0) | 100  (98.2−100) | 100  (98.1−100) |  |

*Abbreviation*: Abbott, SARS-CoV-2 IgG (Abbott); NPA, negative percent agreement; P4D, P4DETECT COVID-19 IgM/IgG (PRIME4DIA); PPA, positive percent agreement; Roche, Elecsys Anti-SARS-CoV-2 (Roche); SARS-CoV-2, severe acute respiratory syndrome coronavirus 2; SDF, STANDARD F COVID-19 IgM/IgG Combo FIA (SD BIOSENSOR); SDQ, STANDARD Q COVID-19 IgM/IgG Combo (SD BIOSENSOR); Siemens, ADVIA Centaur SARS-CoV-2 Total (Siemens).
