# Supplementary material for: Performance of Severe Acute Respiratory Syndrome Coronavirus 2 Serological Diagnostic Tests and Antibody Kinetics in Coronavirus Disease 2019 Patients
Source: Front Microbiol. 2022 Apr 14;13:881038. doi: 10.3389/fmicb.2022.881038 (PMC9048255; doi:10.3389/fmicb.2022.881038)
Supplement: Supplementary file 4 [file Table_4.DOCX]

**Supplementary Table 4.** Sensitivity, specificity, and positive/negative predictive values at 1%, 5%, and 10% seroprevalence of SARS-CoV-2 antibody

| LFIA | COVID-19 | | | | | Sensitivity (95% CI) | Specificity (95% CI) | Seroprevalence | | | | | | |
| --- | --- | --- | --- | --- | --- | --- | --- | --- | --- | --- | --- | --- | --- | --- |
|  | Positive  (n = 389) | |  | Negative  (n = 455)* | |  |  | 1% | 5% | 10% |  | 1% | 5% | 10% |
|  | TP | FN |  | FP | TN |  |  | PPV (95% CI) | | |  | NPV (95% CI) | | |
|  | n = 389* | |  | n = 455^†^ | |  |  |  |  |  |  |  |  |  |
| SDF-IgM/IgG | 250 | 139 |  | 9 | 446 | 64.3% | 98.0% | 24.7% | 63.1% | 78.3% |  | 99.6% | 98.1% | 96.1% |
|  |  |  |  |  |  | 59.3-69.0 | 96.3-99.1 | 14.6-38.6 | 47.1-76.6 | 65.3-87.4 |  | 99.6-99.7 | 97.9-98.4 | 95.6-96.6 |
| SDQ-IgM/IgG | 240 | 149 |  | 12 | 443 | 61.7% | 97.4% | 19.1% | 55.2% | 72.2% |  | 99.6% | 98.0% | 95.8% |
|  |  |  |  |  |  | 56.7-66.6 | 95.4-98.6 | 11.9-29.3 | 41.2-68.4 | 59.7-82.0 |  | 99.6-99.7 | 97.7-98.2 | 95.3-96.3 |
| P4D-IgM/IgG | 219 | 170 |  | 4 | 451 | 56.3% | 99.1% | 39.3% | 77.1% | 87.7% |  | 99.6% | 97.7% | 95.3% |
|  |  |  |  |  |  | 51.2-61.3 | 97.8-99.8 | 19.5-63.3 | 55.9-90.0 | 72.8-95.0 |  | 99.5-99.6 | 97.5-98.0 | 94.8-95.8 |

^*^Included negative samples in the pandemic period of COVID-19. *Abbreviation*: CI, confidence interval; COVID-19, coronavirus disease 2019; FN, false negative; FP, false positive; n, number; NPV, negative predictive value; P4D, P4DETECT COVID-19 IgM/IgG (PRIME4DIA); PPV, positive predictive value; SARS-CoV-2, severe acute respiratory syndrome coronavirus 2; SDF, STANDARD F COVID-19 IgM/IgG Combo FIA (SD BIOSENSOR); SDQ, STANDARD Q COVID-19 IgM/IgG Combo (SD BIOSENSOR); TN, true negative; TP, true positive.
